# A repetitive nucleotide insertion in the *rplV* gene is associated with *in vitro* resistance to azithromycin in *Rickettsia typhi*

**DOI:** 10.1371/journal.pntd.0014249

**Published:** 2026-04-27

**Authors:** Weerawat Phuklia, Kiattawee Chowongkomon, Kaisone Padith, Koukeo Phommasone, Mayfong Mayxay, Allen L. Richards, Elizabeth M. Batty, Matthew T. Robinson, Paul N. Newton, Nicholas J. White, Nicholas P. J. Day, Elizabeth A. Ashley

**Affiliations:** 1 Lao-Oxford-Mahosot Hospital-Wellcome Trust Research Unit, Microbiology Laboratory, Mahosot Hospital, Vientiane, Lao People’s Democratic Republic; 2 Department of Biochemistry, Faculty of Science, Kasetsart University, Bangkok, Thailand; 3 Nuffield Department of Medicine, Centre for Tropical Medicine and Global Health, University of Oxford, Oxford, United Kingdom; 4 Institute of Research and Education Development (IRED), University of Health Sciences, Ministry of Health, Vientiane, Lao People’s Democratic Republic; 5 Naval Medical Research Center, Infectious Disease Directorate, Forest Glen, Maryland, United States of America; 6 Uniformed Services University of the Health Sciences, Preventive Medicine and Biostatistics Department, Bethesda, Maryland, United States of America; 7 Mahidol-Oxford Tropical Medicine Research Unit, Faculty of Tropical Medicine, Mahidol University, Bangkok, Thailand; University of Texas Medical Branch, UNITED STATES OF AMERICA

## Abstract

**Background:**

Murine typhus, caused by *Rickettsia typhi*, is a treatable febrile illness in Laos, where azithromycin treatment failure has been reported. Antibiotic susceptibility testing for *Rickettsia* spp. is challenging due to absence of resistant strains. We aimed to induce an azithromycin-resistant in *R. typhi* and investigate its genetic basis.

**Methodology:**

*R. typhi* Wilmington was cultured in azithromycin-containing media (*R. typhi*^AZM^), starting at a concentration of 0.0019 mg/L and gradually increased to 0.0625 mg/L. Resistant populations were selected up to 0.125 mg/L. MICs were determined using plaque assay and qPCR, and DNA sequencing was performed for *rplD* (L4*), rplV* (L22), and 23S rRNA domain V. Protein modeling of azithromycin-binding sites was conducted, and strain stability was assessed over 24 passages without azithromycin (*R. typhi* ^AZM (-)^).

**Results:**

MICs for wild type (*R. typhi*^WT^) and *R. typhi*^AZM^ were 2 mg/L versus >16 mg/L (plaque assay) and 0.25 mg/L versus 8 mg/L (qPCR). A 15-nucleotides insertion (5’-AAAGGAAGAGCAACT-3’) was found in the *rplV* of *R. typhi*^AZM^, but not other isolates. Protein modeling suggested the insertion extends the L22 loop, potentially affecting azithromycin binding site within the ribosomal exit tunnel. *R. typhi*^AZM^ reverted to wild type MIC and genotype by 24 passages without azithromycin. *R. typhi*^AZM^ exhibited an 8 -fold higher MIC than *R. typhi*^WT^.

**Conclusion:**

Repetitive insertion in *rplV* was associated with azithromycin resistance and may interfere with drug binding*. R. typhi*^AZM^ was unstable without selective pressure. This approach may help generate resistant strains for assay validation. The role of *rplV* mutations in azithromycin susceptibility warrants further investigation.

## Author summary

Murine typhus is a treatable disease, but some patients do not respond well to azithromycin, one of the commonly used antibiotics. Investigating antibiotic susceptibility of *Rickettsia typhi*, the bacteria that causes murine typhus, has been difficult because naturally resistant strains have not been demonstrated conclusively. In this study, we induced an azithromycin-resistant strain of *R. typhi* in the laboratory by gradually exposing it to increasing amounts of the drug. We discovered that resistance was linked to a small genetic change in a ribosomal protein (L22), which likely reduces how well azithromycin can attach to its target. However, when the resistant bacteria were grown without azithromycin, they eventually lost this resistance and returned to their original form. This work provides important insights for understanding treatment failure and for improving laboratory tools to study drug susceptibility.

## Introduction

Murine typhus is an acute febrile illness and a neglected flea-borne disease caused by the obligate intracellular bacterium *Rickettsia typhi*. It is transmitted to humans via the feces of the infected flea being rubbed into bite wounds or mucous membranes [1]. The main vector is the rat flea (*Xenopsylla cheopis*) with rats (*Rattus rattus* and *R. norvegicus*) as the reservoir. Cat fleas (*Ctenocephalides felis*) from domestic cats and opossums (*Didelphis virginiana*) can also transmit this disease [2,3]. Murine typhus has a global distribution except for Antarctica [4]. In Laos, *R. typhi* is an important cause of treatable febrile illness [5] and central nervous system (CNS) infections [6]. A systematic review reported a low mortality rate (0.4%) in untreated patients; however, data from Laos are limited. A prospective study of CNS infection reported mortality of approximately 18% among patients with *R. typhi*/ *Rickettsia* spp., *Orientia tsutsugamushi* and *Leptospira* spp. combined [7]. These data are derived from severe CNS presentations and are not specific to murine typhus alone, and therefore are not representative of overall disease mortality [8]. The main antibiotics used to treat murine typhus include doxycycline, chloramphenicol and azithromycin. Azithromycin, a macrolide antibiotic used to treat various bacterial infections [9], is generally the drug of choice in pregnant women and children aged less than eight years due to concerns that tetracyclines may stain developing bones and teeth, although evidence that doxycycline also leads to staining is lacking [10]. However, a randomised clinical trial in Laos showed significantly more clinical treatment failures among patients with uncomplicated murine typhus treated with azithromycin compared to doxycycline as oral therapy [11].

The cause of these azithromycin treatment failures is unclear, raising concerns about possible differences in susceptibility or emerging resistance to azithromycin in *R. typhi*. Despite its clinical importance, particularly in populations where doxycycline use is limited, the mechanisms of azithromycin susceptibility and resistance in *R. typhi* remain poorly understood. Therefore, this study specifically focuses on azithromycin to investigate the potential factors underlying reduced treatment efficacy. One possible explanation is drug resistance, however assessing antimicrobial susceptibility in *R. typhi* is challenging since this intracellular organism requires host cells for replication and growth [12]. The plaque assay is the gold standard method to measure *Rickettsia* concentration *in vitro* and has been applied to determine antibiotic susceptibility [13,14]. Although this method is able to detect viable *Rickettsia* cultured with and without antibiotics, it is slow and is not able to quantify rickettsia in real time. The incubation period to allow *R. typhi* plaque formation indicating host cell death is 10–13 days, depending on the host cell type [15,16]. Quantitative PCR (qPCR) has been applied as a faster method for MIC determination for obligate intracellular bacteria, including *R. typhi,* for more than twenty years and has the added advantage of permitting quantification of the infecting *in vitro* biomass [17].

Azithromycin acts by inhibiting bacterial protein synthesis targeting the bacterial 50S ribosomal subunit [18]. Mechanisms of azithromycin resistance in different bacteria have been identified and include efflux pump gene expression and ribosomal modification resulting from mutations on the macrolide binding targets [19–21]. There are three major macrolide binding targets, L4 (*rplD*), L22 (*rplV*) and 23SrRNA which are components of a large ribosomal subunit [18]. Mutations on these genes confer macrolide resistance in various bacteria including *Neisseria gonorrhoeae* [22], *Treponema pallidum* [23], *Streptococcus pneumoniae* [24], and *Chlamydia trachomatis* [25]. Culturing bacteria with low concentrations of antibiotic has been applied to study resistance in the obligate intracellular bacterium, *Chlamydia trachomatis* [26].

The aim of this study was to induce azithromycin resistance in the laboratory in the Wilmington strain of *R. typhi* by culturing it with low concentrations of the antibiotic. We evaluated the phenotypic changes by estimating the minimum inhibitory concentration (MIC) of azithromycin in the established strain and comparing it to the wild-type strain (cultured without antibiotic exposure). Additionally, we examined the genotypic characteristics by amplifying the macrolide resistance markers L4 (*rplD)*, L22 (*rplV)*, and 23S rRNA to investigate whether mutations occurred in the DNA sequences of the established strains compared to laboratory strains and clinical isolates.

## Methods

### Ethics statement

These clinical isolates were part of previous studies that were approved by the Lao National Ethics Committee for Health Research (NECHR) and National Institute of Public Health (NIOPH), Vientiane and Oxford Tropical Research Ethics Committee (OxTREC). For all studies, written informed consent was obtained from all adult participants and from parents or legal guardians of child participants prior to sample collection. Studies were approved by the Lao National Ethics Committee for Health Research (NO.25/NECHR) and the Oxford Tropical Research Ethics Committee (024–05).

### Host cell culture

African green monkey kidney cells (VERO; ATCC number CCL-81) were maintained in RPMI 1640 (Gibco, Invitrogen, USA), supplemented with 10% FBS (Sigma Aldrich, USA) and incubated at 35°C in a humidified atmosphere with 5% CO_2_ as described previously [27].

### *Rickettsia typhi* laboratory strains

*R. typhi* strain Wilmington (obtained from the Australian Rickettsial Reference Laboratory, Geelong, Australia), and *R. typhi* laboratory strains AZ306, AZ331, FLA6950, GER, GEAR, PAKNA, MUSSEIBOV and TA837 (obtained from the Naval Medical Research Center (NMRC), Bethesda, USA) were used (Table 1). The *R. typhi* Wilmington strain was used to attempt to establish a resistant strain, and DNA from available laboratory strains was extracted to amplify the macrolide target genes L4 (*rplD)*, L22 (*rplV*), and 23S rRNA for comparison.

**Table 1:** Summary of *Rickettsia typhi* laboratory strains and clinical isolates including strain origin and reference used for macrolide target gene analysis.

Strain/Isolate no.	Origin	Sample source	Year	Reference
Wilmington	North Carolina, USA	Human	1928	Maxcy, 1928 [30]
AZ306	Ethiopia	*Rattus rattus*	1975-6	Univ. Maryland; A.F. Azad [31]
AZ331	Ethiopia	*Rattus rattus*	1975-6	Univ. Maryland; A.F. Azad [31]
FLA H6590	Florida, USA	*Rattus rattus*	1951	Perez Gallardo and Fox, 1948 [32]
Ger	Republic of Georgia	Human	1946	Eremeeva et al,1996 [33]
Gear	South Africa	Human	1939	WRAIR;M.Bozeman (Gear) [34]
Museibov	Republic of Azerbaijan	Human	1949	Eremeeva et al, 1996 [33]
NA18PP (PAKNA)	Pakistan	Rat	1970	Univ. Maryland; C.L. Wisseman, Jr. [31]
TA837	Thailand	*Rattus exulans*	1963	WRAIR; M.Bozeman [31]
TM1041	Vientiane Capital, Laos	Human	2006	Ming et al, 2020 [28]
TM1377	Vientiane Capital, Laos	Human	2007	Ming et al, 2020 [28]
TM2418	Vientiane Capital, Laos	Human	2008	Ming et al, 2020 [28]
TM2504	Xaignabouli Province, Laos	Human	2009	Ming et al, 2020 [28]
TM2522	Vientiane Capital, Laos	Human	2009	Ming et al, 2020 [28]
TM2529	Vientiane Capital, Laos	Human	2009	Ming et al, 2020 [28]
TM2540	Bolikhamxay Province, Laos	Human	2009	Ming et al, 2020 [28]
TM2557	Vientiane Capital, Laos	Human	2009	Ming et al, 2020 [28]
TM3627	Vientiane Capital, Laos	Human	2010	Ming et al, 2020 [28]
TM3905	Vientiane Capital, Laos	Human	2011	Ming et al, 2020 [28]
TM4034	Vientiane Capital, Laos	Human	2012	Ming et al, 2020 [28]
TM4105	Khammouan Province, Laos	Human	2012	Ming et al, 2020 [28]
TM4234	Vientiane Capital, Laos	Human	2012	Ming et al, 2020 [28]
TM4237	Vientiane Capital, Laos	Human	2012	Ming et al, 2020 [28]
TM8956	Vientiane Capital, Laos	Human	2017	Ming et al, 2020 [28]
TM10184	Vientiane Capital, Laos	Human	2019	Ming et al, 2020 [28]

• *Rickettsia typhi* laboratory strains and clinical isolates from Laos that were cultured between 2006 and 2018. Rickettsial group and species were confirmed by IFA and qPCR, respectively. DNA from these isolates was used to amplify the macrolide target genes, L4 (*rplD*), L22 (*rplV*) and 23SrRNA for sequencing. Sequences were compared to those from the established strains (*R. typhi*^AZM^) used for macrolide target gene analysis

### *Rickettsia typhi* clinical isolates

Sixteen stored *R. typhi* clinical isolates (Table 1) were cultured from EDTA blood from patients who participated in studies at the Lao-Oxford-Mahosot Hospital-Wellcome Trust Research Unit (LOMWRU), Vientiane, Lao PDR. These isolates were confirmed for *R. typhi* using an immunofluorescence (IFA) and qPCR [28,29]. In this study, DNA from these isolates were amplified

### *Rickettsia typhi* inoculum preparation

To use the frozen stock of *R. typhi* in short term storage (≤ 2 weeks), the frozen bacteria in Sucrose-Phosphate-Glutamate (SPG) buffer were thawed at 37°C in a water bath; the bacteria were transferred to a 1.5 mL tube and centrifuged at 20,238 xg for 5 min. SPG freezing buffer was discarded and the bacteria were re-suspended in fresh cell culture media and transferred to 2 mL safe-lock microcentrifuge tubes. The cells were lysed using a vortex at maximum speed for 1 min and lysed infected cell suspension was centrifuged at 50 xg for 3 min to separate host cell debris as described in Phuklia et al., 2019 [35]. The supernatant was transferred to a fresh tube and used in the experiment based on the bacterial load (in PFU) determined by plaque assay prior to freezing.

To prepare a fresh stock of bacteria, the flask containing Vero cells infected with *R. typhi*, cultured for 7 days, was scraped using a cell scraper. The suspended cells were transferred to a 2 mL safe-lock microcentrifuge tube [36]. The processes of cell lysis and bacterial separation were described above. The supernatant was transferred into a fresh tube and used in the experiment. To determine the bacterial load from the fresh culture, qPCR was used.

### *Rickettsia typhi* cultured under low concentration of azithromycin

Monolayers of Vero cells were inoculated with *R. typhi* (Wilmington) and cultured in 2% FBS in RPMI, either with a sub-MIC concentration 0.0019 mg/L of azithromycin (analytical standard, Sigma-Aldrich, UK; Cat. No. 75199), labelled as *R. typhi*^AZM^, or without (*R. typhi*^WT^) to act as a control. The drug concentration in *R. typhi*^AZM^ was increased 2-fold every four passages or every month depending on the cytopathic effect (CPE) observed compared to the control (*R. typhi*^WT^). The CPE in *R. typhi*^WT^ and *R. typhi*^AZM^ was assessed by cell clumping (S1 Fig) visualized by microscopy. *R. typhi*^AZM^ was maintained in culture media containing azithromycin concentrations up to 0.0625 mg/L for two years as illustrated in S2 Fig. Antibiotic susceptibility testing using the plaque assay was then determined as described below. A plaque is an area of host cells destroyed by infecting bacteria seen as a clear zone (damaged cells) around a dark zone (healthy cells) as illustrated in Fig 2B. MIC was determined as the lowest concentration of antibiotics that inhibited plaque formation.

**Fig 1:**
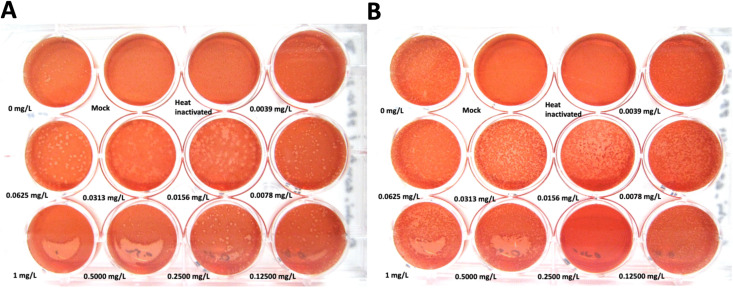
Azithromycin susceptibility testing using the plaque assay for *R. typhi* wild type strain, *R. typhi*^WT^ and *R. typhi* strains under antibiotic pressure, *R. typhi*^AZM^ (A and B). Azithromycin concentrations ranging from 0.0039 to 1 mg/L were used to determine the MIC for *R. typhi*^WT^ (**A**) and *R. typhi*^AZM^ **(B)**. Plaque formation with no antibiotic was used as a positive control and heat-inactivated bacteria (56°C for 30 min) were used as a negative control to show that the bacteria were not able form plaques. The mock well, which did not contain rickettsia, was used as a control for host cell morphology during long-term incubation (14 days) to confirm that the cells could survive until the time of evaluation. MIC was determined as the lowest concentration of antibiotic at which rickettsia was not able to form plaques. MIC for *R. typhi*^WT^ as 1 mg/L whereas for *R. typhi*^AZM^ it was > 1 mg/L. Images shown represent a single well from a representative plate at each azithromycin concentration. Experiments were performed independently with three replicates on separate plates, and consistent plaque phenotypes were observed across all replicates.

To select the antibiotic-resistant *R. typhi* population from the culture, *R. typhi* plaques that survived in media containing antibiotics at sub-inhibitory concentrations for plaque formation (*R. typhi*^AZM^) were selected along with plaques from the culture without azithromycin as the control (*R. typhi*^WT^) using a pipette tip (S2 Fig). The plaque in the agarose gel was picked using a micropipette tip and mixed with 0.9 mL of fresh media. The mixture of plaque and media was inoculated into new host cells. The culture in the flask with azithromycin was maintained in media containing 0.0625 mg/L of azithromycin for four generations to ensure the bacteria could still grow under these conditions before increasing the concentration to 0.125 mg/L and keeping in culture until determination of MIC.

### Plaque assay

The plaque assay was used to detect the viability of *Rickettsia* as previously described [14,37,38]. Briefly, Vero cells were plated in a 12-well plate overnight. Two hundred microliters of *R. typhi* inoculum was inoculated to the overnight monolayer of Vero cells. Infected cells were incubated at 35°C with 5% CO_2_ for 1.5 hr. The plate was rocked and rotated every 15 min during the incubation period. To prepare 2X RPMI for the plaque assay, the final concentration of FBS was adjusted to 8%. Agarose was then added by combining equal parts of 1% agarose solution and plaque media, resulting in final concentrations of 0.5% agarose, 4% FBS, and 1X RPMI in the overlay medium. The plate was incubated at 35°C with 5% CO_2_ for 14 days. At day 12, 0.01% neutral red in PBS solution was added to each well and incubated overnight. The neutral red was discarded at day 13. Plaque formation was observed and counted at day 14.

Crystal violet was also used for plaque staining in the antibiotic susceptibility assay. The procedures for inoculation and incubation were the same as described above. However, the media for plaque assay was mixed with sterile 0.6% Avicel in distilled water. The final concentration of the overlay media for crystal violet staining was 0.3% Avicel with 4% FBS in 1X RPMI. The plate was incubated at 35°C with 5% CO_2_ for 14 days. At day 14, ice cold methanol was applied for 1.5 h for fixing. Then, the methanol was discarded and 1% crystal violet in 20% ethanol was used for staining. The crystal violet was discarded and the stained plates were rinsed with tap water.

### Antibiotic susceptibility testing assay

#### MIC determination by plaque assay.

To determine the MIC of azithromycin for *R. typhi*^WT^ and *R. typhi*^AZM^, an inoculum from each culture type was prepared as described above and added to each well. In the 12-well plate assay, 10 wells were inoculated with *R. typhi* strains to be tested, and the remaining two wells were used as controls: one without *Rickettsia* as the host cell (Vero) control and one with heat-inactivated *Rickettsia* (56°C for 30 minutes) as a negative control to calculate the MIC as there was no plaque formation. The inoculated plate was incubated at 35°C for 1.5 hr; the plate was rocked and rolled every 15 minutes during the incubation period. Then, the plaque media with and without serial dilutions of azithromycin were added. The concentration range of azithromycin between 0.0625 – 16 mg/L was used to determine the MIC for the bacteria. The plate was incubated as above according to the type of the mixture (agarose or Avicel). MIC using plaque assay was determined as the lowest concentration of azithromycin that inhibited *Rickettsia* plaque formation, after 14 days incubation. All plaque assays were performed in independent experiments using separate plates for each azithromycin concentration. For each condition, three replicates were included, and consistent plaque phenotypes were observed across replicates. Representative images from a single well of a representative plate are shown in the figures.

#### MIC determination by qPCR.

To determine the MIC based on inhibition of bacterial DNA synthesis, antimicrobial susceptibility testing (AST) was performed using the same infection procedure as the plaque assay. However, instead of quantifying plaque formation, bacterial growth was measured by qPCR, with a different incubation period used for endpoint determination. Cell infection at day 0 was used to compare with *R. typhi* infection at day 7, and heat-inactivated *R. typhi* was used as the negative control to estimate the MIC. Cultures were incubated for 7 days before harvesting by trypsinization. The infected Vero cells were pelleted by centrifugation at 20,238 xg for 5 min. The media was discarded and the infected cell pellet were kept at -80°C until used. DNA was extracted using the HotShot method previously applied to extract DNA from obligate intracellular bacteria [36]. Briefly, the infected cell pellet was resuspended in 50 μL of alkaline lysis buffer (25 mM NaOH, 0.2 mM EDTA) and boiled at 95°C for 30 min. The sample was cooled to 4°C and an equal volume (50μL) of neutralization buffer (40 mM Tris-HCl, pH 7–8) was added. Extracted DNA from each antibiotic condition, including untreated controls and heated-inactivated samples was subjected to qPCR targeting *ompB* gene. The primers and TaqMan probe used were Rt557F (5′-TGGTATTACTGCTCAACAAGCT-3′), Rt678R (5′-CAGTAAAGTCTATTGATCCTACACC-3′), and Rt640 BP (5′-FAM-CGCGATCGTTAATAGCACCAGCATTATCGCG-BHQ1–3′). The qPCR reaction mixture consisted of 1X qPCRBIO Probe Mix (qPCR Probe MIX LO-ROX, PCR Biosystems, UK), 0.4 μM of each forward and reverse primer, 0.2 μM probe, sterile distilled water, and 1 μL of extracted DNA. Amplification was performed using a CFX96 real-time PCR system (Bio-Rad, USA) under the following conditions: initial denaturation at 95°C for 2 min, followed by 45 cycles of denaturation at 95°C for 15 s and combined annealing/extension at 60°C for 30 s, with fluorescence acquisition at each cycle. MIC determination by qPCR was defined as the lowest azithromycin concentration corresponding to a cycle threshold (Ct) value equal to or higher than that of the heat-inactivated sample. As these MIC values represent threshold rather than continuous measurements, statistical analysis was not performed. Experiments were conducted in three independent replicates with consistent results.

### Plaque selection

To identify probable azithromycin resistant populations, plaques forming under azithromycin treatment were picked for the isolation of homogenous azithromycin resistant *R. typhi*. The method was adapted from virus purification by plaque assay [39]. Briefly, a sterile pipette tip was used to penetrate the agar containing the plaque. The tip with agar was transferred to a microcentrifuge tube containing the media and the plaque was mixed by pipetting. The mixture was inoculated to a new flask for propagation of *R. typhi*.

### PCR and sequencing

DNA from the *R. typhi* cultured under azithromycin, *R. typhi* clinical isolates and *R. typhi* laboratory strain cultures were extracted as described in manufacturer’s instructions (ThermoFisher Scientific, US). *R. typhi* growing for a week after thawing from frozen stock (*R. typhi*^low passage^) was also used for comparison. Briefly, infected cells were scraped using a cell scraper (Corning, US) and centrifuged at 20,238 xg for 5 min to get the cell pellet. The cell pellet was resuspended with 200 μL of phosphate buffer saline (PBS) in a microcentrifuge tube containing 20 μL of proteinase K, then 400 μL of lysis buffer was added and the tube incubated at 56°C for 15 min. The mixture was precipitated with absolute ethanol before transfer to the Spin Column. The mixture was spun and washed to remove the unbound DNA. DNA was eluted using elution buffer 100 μL and kept at minus 20°C until used. DNA from these isolates was amplified using 23SrRNA, L4 and L22 primers for sequencing (S1 Table). The PCR products were run on 1.5% agarose gel for visualization, and the PCR products were purified using the GeneJet PCR Purification Kit (ThermoFisher Scientific, US) before sending the samples for Sanger sequencing to MACROGEN (Seoul, South Korea).

### Sequence analysis

All DNA nucleotide sequences in ABI format were edited according to the chromatogram using DNA star Lasergene 17 (USA). The edited nucleotide sequences were aligned using MEGA11 to compare the mutations among the sequence and reference sequence obtained from the Kyoto Encyclopedia of Genes and Genomes (KEGG).

### Protein homology

The 3D structure of the L4 and L22 proteins for *R. typhi* were modeled using the SWISS-MODEL server with default settings. The translated protein sequences from DNA sequence data were entered in FASTA format, and 3D homology models were retrieved as PDB files for visualization.

The 3D structures of azithromycin binding protein targets (L4, L22) and 23SrRNA were searched in the Protein Data Bank (PDB: https://www.rcsb.org). The crystal structure of azithromycin bound to the G2099A mutant 50S ribosomal subunit of *Haloarcula marismortui* (1YHQ) was used as a template to create the azithromycin-target complex (L4, L22, and 23S rRNA) by superimposing *R. typhi* L4 and L22.

We used Discovery Studio Visualizer to explore the L22 protein model constructed from amino acid sequences of *R. typhi*^AZM^ and *R. typhi*^WT^

## Results

### *In vitro* screening for azithromycin resistance

After culturing *R. typhi* in Vero cells with media containing a low concentration of azithromycin for 78 weeks, the MIC of *R. typhi*^AZM^ for azithromycin was determined using the plaque assay and compared to *R. typhi*^WT^. The results showed MIC of azithromycin for *R. typhi*^WT^ was 1 mg/L (Fig 1A) and MIC for *R. typhi* ^AZM^ was greater than 1 mg/L (Fig 1B).

### Selection of azithromycin resistant *Rickettsia typhi*

**Fig 2:**
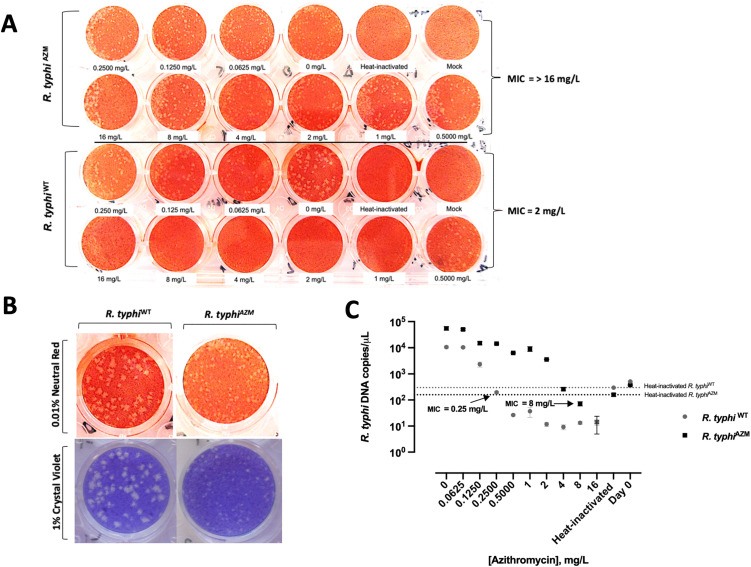
MIC of azithromycin determined by plaque assay and qPCR for *R.* *typhi*^WT^ and *R. typhi*^AZM^. Antibiotic susceptibility testing based on plaque forming units was performed to determine MIC for *R. typhi*^WT^ and *R. typhi*^AZM^ **(a)**. MIC of azithromycin for *R. typhi*^WT^ was 2 mg/L (**A**, lower panel) and MIC for *R. typhi*^AZM^ was > 16 mg/L (**A**, upper panel). Different plaque sizes obtained from *R. typhi*^WT^ (**B**, left panel) and *R. typhi*^AZM^ (**B**, right panel). *R.* *typhi*  plaques were stained with 0.01% neutral red in PBS (b, upper panel) and 1% crystal violet in 20% ethanol in distilled water (**B**, lower panel). Images shown represent a single well from a representative plate at each azithromycin concentration. Experiments were performed independently with three replicates on separate plates, and consistent plaque phenotypes were observed across all replicates. MIC of azithromycin based on the Rickettsial DNA load of *R. typhi*^WT^ and *R. typhi*^AZM^ was determined at various concentrations of azithromycin using qPCR targeting ompB gene **(C)**. The x-axis represents azithromycin concentration ranging between 0.0625 – 16 mg/L and the y-axis represents *R. typhi* DNA (copies/μL). Each dot on the graph represents rickettsia DNA load in various concentrations of azithromycin including no antibiotic as a positive control. Grey dots represent rickettsial DNA from wild type strain and black squares represent rickettsia DNA from strains under drug pressure. Heat-inactivated bacteria were used to determine the MIC (grey dotted line and black dotted line), and bacterial DNA load on Day 0 measured to confirm the bacteria growth. MIC of azithromycin for *R. typhi*^WT^ (grey) was 0.25 mg/L and MIC for *R. typhi*^AZM^ (black) was 8 mg/L. The MIC for drug-pressured strains was higher than wild type strains by about 32-fold. MIC values were defined as the lowest concentration inhibiting detectable bacterial growth and therefore represent threshold rather than continuous measurements; accordingly, no statistical analysis was performed. Experiments were conducted in three independent replicates with consistent results.

To select the azithromycin-resistant population, the *R. typhi*^AZM^ plaque (Fig 1B) visible at a concentration of 0.125 mg/L was picked for inoculation to the new flask containing media with 0.125 mg/L of azithromycin. After the bacteria was cultured for a month, antibiotic susceptibility testing was performed for *R. typhi*^AZM^ and *R. typhi*^WT^ in paralllel. Fig 2A shows plaque formation at different concentrations of azithromycin ranging from 0.0625 mg/L to 16 mg/L. Inhibition of *R. typhi*^WT^ was observed at 2 mg/L (Fig 2A, lower panel) while *R. typhi*^AZM^ plaques were able to form at a concentration of 16 mg/L (Fig 2A, upper panel) indicating that the MIC for *R. typhi*^AZM^ was > 16 mg/L while the MIC for *R. typhi*^WT^ was 2 mg/L. The plaque size of *R. typhi*^AZM^ (Fig 2B, right panel) was observed to be smaller compared to *R. typhi* ^WT^ (Fig 2B, left panel).

MIC of azithromycin was also determined by qPCR. MIC for *R. typhi*^WT^ was calculated to 0.25 mg/L whereas the MIC of azithromycin for *R. typhi*^AZM^ was 8 mg/L, as illustrated in Fig 2C.

### Mutation in *rplV* gene of *Rickettsia typhi* with azithromycin

**Fig 3:**
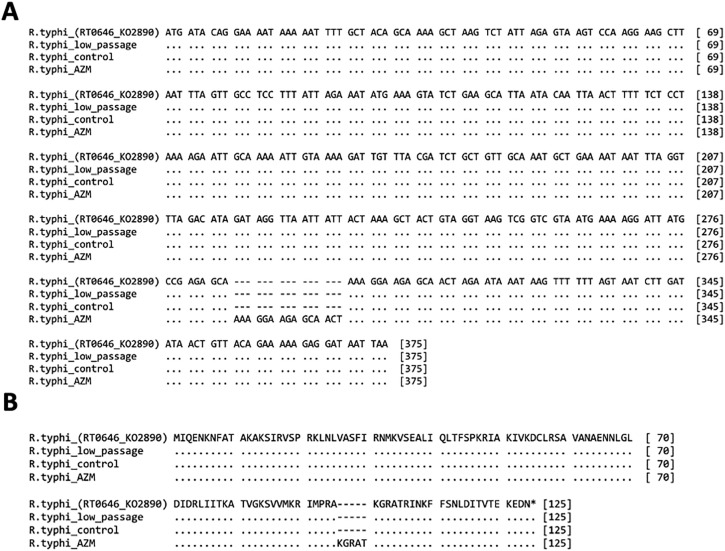
Sequence alignment for L22 (rplV). DNA sequence (**A**) and amino acids sequence (**B**) from various conditions of *R. typhi* culture; *R. typhi* low passage = *R. typhi* growing for a week after thawing from frozen stock, *R. typhi* _control (*R. typhi*^WT^) = *R. typhi* culture without azithromycin. *R. typhi* _AZM = *R. typhi* cultured with low concentration of azithromycin for long period (90 generations). These DNA and amino acid sequences were compared with reference DNA sequences from Kyoto Encyclopedia of Genes and Genomes (KEGG).

The sequencing results showed fifteen nucleotide insertions (5’-AAAGGAAGAGCAACT-3’) in the *rplV* DNA sequence for *R. typhi*^AZM^ but not *R. typhi*^WT^ and *R. typhi*^low passage^ (Fig 3A). *R. typhi*^low passage^ represent *R. typhi* grown for one week after thawing from frozen stock. The reference sequence of *R. typhi* strain Wilmington did not contain this sequence insertion. Mutation was not observed on *rpLD* and *23SrRNA* DNA sequence for all strains (S3 Fig). The protein sequence of L22 (*rplV*) was translated from the nucleotide sequence using MEGA11, revealing the insertion of five amino acids (96KGRAT100) (Fig 3B). These data suggested that a 5 amino acids insertion on the L22 protein may interfere with azithromycin binding.

Translated L22 amino acid sequences from other *R. typhi* clinical and laboratory isolates were also used for comparison with *R. typhi*^AZM^. No mutation for the L22 protein was detected in those isolates (S4 Fig).

### L22 protein in wild type and mutant *Rickettsia typhi*

**Fig 4:**
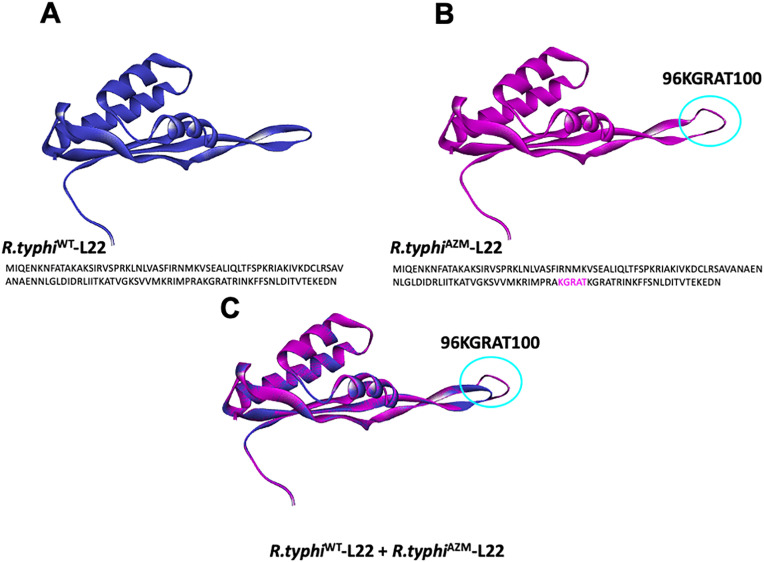
L22 ribosomal protein model of *R. typhi* wild type, *R. typhi* ^WT^ (A) and *R. typhi* with azithromycin, *R. typhi* ^AZM^ (B). The model no. B0BUQ5.1.A, large ribosomal subunit protein L22 from *R. rickettsia* (strain Iowa) was used as a template to construct the model. The extended loop (light blue circle) in the mutant model (B) represents the insertion of repeat 5 amino acid KGRATK. Superposition between L22_*R. typhi*^WT^and L22_*R. typhi*^AZM^ **(C)**.

To explore the impact of the insertion of five amino acids on the L22 protein sequence on *R. typhi*^AZM^ azithromycin binding, the sequence with and without the amino acids insertion was modeled. The *R. typhi* L22 for both strains were based on a template of *Rickettsia rickettsii* strain Iowa (BOBVQS5.1A) which has 89.92% sequence identity and covers 100% of the L22-*R.typhi* protein sequence. The L22 models from the protein sequence of *R. typhi*^WT^ (without insertion) (Fig 4A) and L22 protein with 5 amino acids insertion (96KGRAT100), *R. typhi*^AZM^, were constructed (Fig 4B). An extended loop in L22 with amino acids insertion (light blue circle) was identified but was not present in L22 wild type. The protein of the wild-type strain was overlaid with the mutant protein (Fig 4C). This extended loop changed the protein’s structure of L22 compared to the wild type.

### Homology modeling of azithromycin protein binding

**Fig 5:**
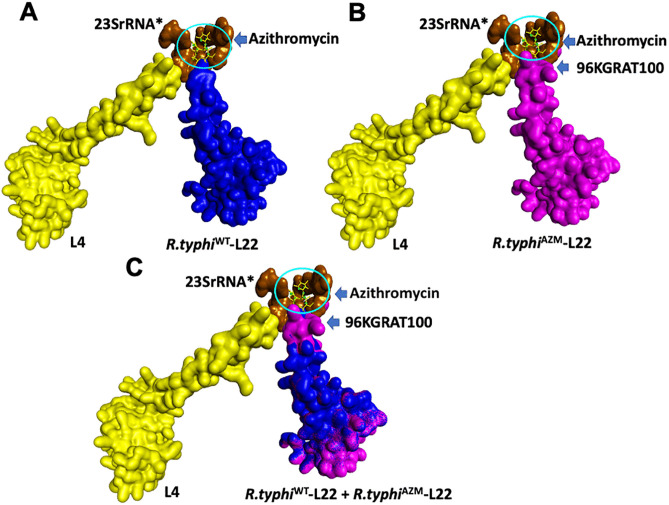
Binding interaction of azithromycin and its key ribosomal targets in *R. typhi.* Azithromycin (yellow ring structure) interacts with ribosomal proteins L4, L22, and specific nucleotides of 23S rRNA. The *R. typhi* L4 protein (yellow) was modeled using the L4 template from *R. prowazekii* strain Madrid E (Q9ZCQ6.1.A). A partial 23SrRNA segment (brown) was modeled based on the 50S ribosomal subunit of Haloarcula marismortui (PDB ID: 1YHQ). Two models of *R. typhi* L22 were constructed using the *R. rickettsii* strain Iowa template (BOBVQS5.1A): one without a 5-amino acid insertion (blue surface) and one with the insertion (magenta surface). Panels (A) and (B) show the interaction of both L22 variants with L4, azithromycin, and key 23S rRNA nucleotides. Panel (C) shows the merged model of L22 from *R. typhi*^WT^ and *R*. *typhi*^AZM^, highlighting an extended loop at the azithromycin binding site (blue arrow). Key 23S rRNA nucleotides involved: A2099, A2100, A2103, A2538, G2540, U2645 and G2646. 23S rRNA* represents the partial nucleotide sequence involved in interactions with L4, L22, and azithromycin.

The molecular simulation of azithromycin and its targets interaction for *R. typhi* was predicted based on the 3D structure of azithromycin bound to the 50S ribosomal subunit of *Haloarcula marismortui* (PDB ID: 1YHQ). L4, L22 and 23SrRNA were the focus of study of azithromycin binding interactions and other large protein subunits of the complex were deselected. The constructed *R. typhi*-L4 protein was modeled based on the L4 protein template of the *R. prowazekii* strain Madrid E (Q9ZCQ6.1.A) with 94.69% sequence identity and 100% coverage (Fig 5, yellow surface molecule. The constructed *R. typhi*-L22 protein without insertion (*R.typhi*^WT^-L22) (Fig 5A, blue surface molecule) and with insertion (*R.typhi*^AZM^-L22) (Fig 5B, magenta surface molecule) were modeled based on L22 protein template of *Rickettsia rickettsii* strain Iowa (BOBVQS5.1A). The surface model of the azithromycin binding mechanism with the protein targets showed that the insertion of 5 amino acids in the L22 protein might obscure the azithromycin binding pocket (Fig 5B) compared to L22 from *R.typhi*^WT^ (Fig 5A). Superimposed images of *R.typhi*^WT^-L22 and *R.typhi*^AZM^-L22 (Fig 5C) revealed that the extended loop of L22 protein from *R. typhi*^AZM^ interferes with the drug binding pocket leading to reduced susceptibility to azithromycin.

### Stability of azithromycin-resistant *Rickettsia typhi*

To investigate the stability of azithromycin resistance in *R. typhi*^AZM^, the strain was passaged in Vero cells in the absence of azithromycin (designated *R. typhi*^AZM(-)^), and the MIC of azithromycin was determined at the 10^th^ (10 weeks) and 24^th^ (24 weeks) generations. These values were compared with those of *R. typhi*^AZM^ and *R. typhi*^WT^ as described in Table 2. For the 24^th^ generation*. R. typhi* Wilmington (low passage) was included for comparison.

**Table 2:** MIC determination of azithromycin for *Rickettisia typhi* strains using a plaque assay over 24 generations.

Number of generation/ Culture condition	MIC of azithromycin (mg/L)			
	*R. typhi* ^low passage^	*R. typhi* ^WT^	*R. typhi* ^AZM^	*R. typhi* ^AZM(-)^
Generation 0	NA	2	>16	NA
10 generations (10 weeks)	NA	1	8	4
24 generations (24 weeks)	0.125	0.25	8	0.25

NA = Not assessed.

Plaque assay results showed that, at the 10^th^ generation, the MIC of *R. typhi* ^AZM(-)^ decreased to 4 mg/L compared with >16 mg/L in the starting *R. typhi*^AZM^ population. After 24 generations, the MIC of *R. typhi*^AZM(-)^ further decreased to 0.25 mg/L, which was comparable to that of *R. typhi*^WT^.

At the 10^th^ generation, the MIC of *R. typhi*^AZM^ (8 mg/L) was 2-fold higher than that of *R. typhi*^AZM(-)^ (4 mg/L). At the 24^th^ generation, the MIC of *R. typhi*^AZM^ (8 mg/L) remained 32-fold higher than that of *R. typhi*^AZM(-)^ (0.25 mg/L).

We also investigated the *rplV* (L22) DNA sequences using *R. typhi*^low passage^, *R. typhi*^WT^, *R. typhi*^AZM^ and *R. typhi*^AZM(-)^. The result showed that insertion of fifteen nucleotides or five amino acids were not found in *R. typhi*^AZM(-)^ and the DNA sequence was conserved with *R. typhi*^low passage^ and *R. typhi*^WT^ whereas fifteen repetitive nucleotides was still found in the *R. typhi*^AZM^ (S5 Fig).

## Discussion

This is the first report of the establishment of an azithromycin resistant strain of *Rickettsia typhi* by long term culture with a low concentration of antibiotic [40]. This drug pressure approach has been used with another obligate intracellular bacteria, such as *Chlamydia trachomatis,* by selecting the wild type bacteria from the repeated passage of the surviving bacterial culture in the presence of sub-inhibitory doses of erythromycin, azithromycin and josamycin [26]

Observation of the cytopathic effect of *R. typhi*^AZM^ compared with *R. typhi*^WT^ was able to demonstrate that *R. typhi*^AZM^ can replicate under antibiotic pressure. After long term culture for 2 years in media containing low concentrations of azithromycin, *R. typhi*^AZM^ was able to grow at a concentration of 0.0625 mg/L, approximately 32-fold higher than the first concentration. Moreover, the difference of plaque size between *R. typhi*^WT^ and *R. typhi*^AZM^ might be useful to distinguish between susceptible and resistant strains. The observed difference in plaque morphology between wild-type and azithromycin-resistant *R. typhi* strains may also reflect underlying differences in bacterial fitness and virulence. Although this was not directly assessed in the present study, future work should investigate key aspects of the infection process, including bacterial entry into host cells and intracellular replication rates, particularly in relevant human endothelial cell models. In addition, *in vivo* studies would be valuable to determine whether differences in plaque size correlate with altered virulence between strains.

Plaque purification is a method used to ensure the homogeneity of the bacteria stock [39]. This approach was applied to select the resistant population of *R. typhi* that survived under antibiotic treatment. The result showed more than eight-fold difference in MIC between *R. typhi*^WT^ and *R. typhi* ^AZM^. MIC testing by qPCR suggested a 32-fold difference in susceptibility level between the two strains. However, the MIC values determined by plaque assay and qPCR showed differences (*R. typhi* ^WT^: 2 mg/L (plaque assay) vs. 0.25 mg/L (qPCR); *R. typhi* ^AZM^: > 16 mg/L (plaque assay) vs. 8 mg/L (qPCR)). This difference might be explained by the fact that qPCR detects both live and dead bacteria, and heat-inactivated *Rickettsia* was used for the MIC determination via qPCR. As a result, it appears that lower concentrations can inhibit *R. typhi* growth. In contrast, the plaque assay measures viable *Rickettsia*, requiring higher concentrations to inhibit plaque formation.

Subsequently *R. typhi*^*AZM*^ was cultured in RPMI without azithromycin (*R. typhi*^AZM(-)^) for 24 generations. We found the MIC for azithromycin for *R. typhi*^AZM(-)^ was lower than the 0 generation but was still higher than the *R. typhi*^WT^ and *R. typhi* low passage for the first 10 generations. However, by the 24^th^ generation, the MIC of azithromycin for *R. typhi*^AZM(-)^ was the same as the MIC of azithromycin for wild type strains and the low passage strains. These findings suggest that azithromycin resistance in *R. typhi* is not heritable and may reflect the presence of mixed population, in which the less fit resistant subpopulation was eliminated in the absence of selective pressure.

Azithromycin is a macrolide antibiotic which inhibits bacterial protein synthesis via the 50S large subunit of the bacterial ribosome. Efflux pump activation and modification of antibiotic target are mechanisms that the bacteria use to escape antibiotic effects [18]. The main antibiotic target gene for macrolide antibiotics including azithromycin is Domain V of *23SrRNA*, L4 (*rpl*D) and L22 (*rplV*). Studies of *C. trachomatis* culture in McCoy cells in the presence of erythromycin, azithromycin and josamycin have shown mutation on L4 (*rplD*) and the domain V of *23SrRNA* but not L22 (*rplV*) [26]. However, in the investigation of macrolide target genes among clinical isolates, a mutation in the L22 (*rplV*) gene was found. Despite this, the MICs for the three antibiotics were the same as those for the *C. trachomatis* reference strain, except in isolates with mutations in the 23S rRNA or both the 23S rRNA and L22 genes, which showed higher MICs compared to the reference strain [41]. In this study, macrolide sequence data of *R. typhi*^*A*ZM^ showed the insertion of a repetitive 15 nucleotide sequence (5 amino acids) on the L22 protein (*rplV*). However, we did not see any mutation on L4 (*rplD*) and Domain V of 23SrRNA nucleotide sequence. Our findings are similar to those from *S. aureus* cultured under sub-inhibitory concentrations of erythromycin (8325^ER+^) which showed a 27 nucleotide repeat insertion (9 amino acids insertion) in the *rplV* gene that encodes the L22 protein, conferring resistance to macrolide antibiotics [42], and to tylosin susceptibility in *S. xylosus* [43]. Aside from comparing the sequence with the reference strain and *R. typhi* Wilmington, we also compared the L22 (*rplV*) sequence of *R. typhi*^AZM^ with seven laboratory strains and sixteen Lao clinical isolates. Insertion in the L22 (*rplV*) sequence was not found in all 23 isolates. These results suggest that azithromycin selective pressure on *R. typhi* might have led to the selection of a strain containing a repeat nucleotide sequence.

Molecular dynamic simulation is a useful tool to predict the interaction between molecules and has been to applied to drug discovery to explore the binding efficiency between drug and target [44]. This approach was used to investigate whether the insertion of the repeated amino sequence in the L22 protein of *R. typhi*^AZM^ would affect azithromycin binding. We found that insertion of a repeated amino acid sequence in the L22 protein of *R. typhi*^AZM^ affects the structures compared to wild type bacteria. The macrolide binding pocket is composed of the loop of the L22 protein, the loop of the L4 protein and specific nucleotides in the domain V region of 23S rRNA form a pocket for azithromycin binding. The extended loop resulting from amino acid insertion of *R.typhi*^AZM^ interferes with binding of azithromycin to the pocket. Our findings suggest that the insertion of repeated sequence in the L22 protein in *R. typhi*^AZM^ might affect the drug binding site leading to lower susceptibility to azithromycin compared to wild type.

Azithromycin-resistance in *R. typhi* was not heritable when drug pressure was removed and *R. typhi* ^AZM^ was cultured under normal conditions (culture media without antibiotic) for 24 generations. The MIC reverted to 0.25 mg/L which was the same value as *R. typhi* ^WT^ and similar to *R. typhi* low passage. This might be explained by the processes governing reversal of antimicrobial-drug resistance in bacterial populations after removal of selective pressure. Firstly, fitness cost reduces the frequency of resistance. Secondly, the biological cost of resistance may be decreased over time through compensatory mutations that reduce the rate at which resistant clones are outcompeted by susceptible counterparts. Lastly, selective pressure within the population may prevent the reversion to a susceptible phenotype by maintaining the resistant subpopulation. These events occur gradually over time with the number of bacterial passages [45]. The MICs of azithromycin for both *R. typhi* ^WT^ (0.25 mg/L) and *R. typhi* ^AZM^ (8 mg/L) of the 24^th^ generation were different from the earlier experiment (*R. typhi*^WT^ = 2 mg/L and *R. typhi* ^AZM^ = > 16 mg/L). These differences may reflect minor experimental variability, including host cell conditions used for plaque assays. In addition, both the number of bacterial passages and host cell passages may influence antibiotic susceptibility. These findings were different from the study on *Chlamydia trachomatis* and *S. aureus*. Resistance was heritable and the resistance persisted even when cultured without antibiotic [26,42]. As azithromycin is a bacteriostatic drug, some wild type strains of the bacteria may still survive but not replicate in the culture with a low concentration of antibiotics. Once azithromycin was removed from the culture, the wild type population was able to replicate and overcome the replication of *R. typhi*^AZM^. Future work should conduct competitive assay experiments between *R. typhi*^WT^ and *R. typhi*^AZM^ under normal culture conditions to further investigate *R. typhi*^WT^ and *R. typhi*^AZM^ populations.

This study was undertaken following clinical observation of treatment failure following three days of azithromycin treatment in patients with murine typhus reported by Newton et al. [11]. Rickettsia isolates from that study were not submitted to *in vitro* susceptibility testing or sequencing. To date, no specific genetic markers of azithromycin resistance have been reported in *Rickettsia* spp. Previous *in silico* analyses of the ribosomal protein L22 across typhus group (TG) species (*R. typhi* and *R. prowazekii*) and several spotted fever group (SFG) species (e.g., *R. africae*, *R. rickettsii*, *R. conorii*) have identified mutations in SFG but not in TG species. These findings suggest that intrinsic differences in macrolide susceptibility may exist between groups, with SFG potentially exhibiting reduced susceptibility to macrolides such as erythromycin [46]. Taken together, our findings likely represent early evidence of potential resistance-associated mutations identified under laboratory conditions. Further epidemiological surveillance and validation in clinical isolates are required to determine whether these genotypes are present and clinically relevant.

Although further validation is required, these findings may have important clinical implications. Doxycycline is currently the first-line treatment for murine typhus, while azithromycin is commonly used as an alternative, particularly in pregnant women. However, a clinical trial in Laos demonstrated that patients with murine typhus treated with azithromycin experienced prolonged fever clearance times compared to those treated with doxycycline [11]. As *Rickettsia typhi* is an obligate intracellular bacterium, conventional antibiotic susceptibility testing methods, such as microdilution and disk diffusion, are not applicable. Although cell culture–based susceptibility assays can be performed [17,47], they are labor-intensive and time-consuming. Therefore, the identification of genetic markers associated with azithromycin resistance has important clinical implications. These markers could be developed into molecular diagnostic tools to rapidly detect resistance in clinical samples, thereby guiding clinicians in selecting the most appropriate antibiotic therapy and improving patient outcomes.

There were some limitations of this study. Firstly, we investigated antibiotic pressure only for *R. typhi* strain Wilmington; different bacterial isolates might vary in their response to the antibiotic. Secondly, other mechanisms of antibiotic resistance, including efflux pump activation, were not investigated. Although whole-genome sequencing was not performed for the induced azithromycin-resistant isolates in this study, we interpreted our findings in the context of previously available *Rickettsia typhi* genomes from our earlier work [48]. These analyses demonstrated extremely low genetic diversity across isolates collected over nearly a century and from multiple continents, with only limited temporal clustering and minimal evidence of host-specific adaptation. Importantly, no mutations were identified in macrolide resistance-associated loci, including *rplV* (L22), *rplD* (L4), or the 23S rRNA gene, in these datasets. Consistent with this, resistance-associated mutations appear to be rare in *R. typhi*, suggesting strong evolutionary constraint. In this study, we identified a nucleotide insertion in the L22-encoding gene (*rplV*) in an induced azithromycin-resistant strain. To our knowledge, this mutation has not been observed in previously sequenced *R. typhi* genomes, suggesting that it may represent a novel adaptation under antibiotic pressure. However, without whole-genome comparison of the induced strain and functional validation, the contribution of this mutation to azithromycin resistance cannot be definitively established. Thirdly, the crystal 3D structure of the L22 protein including L4 and 23S rRNA for *R. typhi* were not available in the database, so we had to predict the structure from the related species, *Rickettsia rickettsii* for L22, and *Rickettsia prowazekii* for L4. This might have led to some errors in identifying the antibiotic binding interaction. Finally, azithromycin was added at the start of the experiment and not replenished during the 7-day incubation period. Consequently, the effective drug concentration may have decreased over time due to potential degradation at 35 °C, and the measured MIC values likely reflect cumulative rather than constant exposure. This may lead to underestimation of the true MIC; however, as all experiments were performed under identical conditions, comparisons between wild-type and azithromycin-resistant *R. typhi* strains remain valid.

Further work, including whole-genome sequencing of the induced resistant isolates and functional validation studies, is required to determine whether additional genomic changes contribute to azithromycin resistance. In addition, validation using independent clinical isolates will be important to assess whether similar genotypic and phenotypic characteristics occur in natural settings.

Our findings suggest mutation in *rplV* could modulate azithromycin resistance in *R.typhi* and highlight the need for validated antibiotic susceptibility testing assays. Further investigation in clinical infections is needed.

## Supporting information

S1 Table Macrolide target genes including Domain V of 23SrRNA (DomV), L4 and L22 primers. (DOCX)

S1 Fig Host cell morphology during *R. typhi* infection. Vero cells were infected with *R. typhi* strain Wilmington in the culture media without azithromycin (B) and in the low level of azithromycin (C) compared to host cell control (A). CPEs were found both *R. typhi*^WT^ and *R. typhi*^AZM^ at Day 7 post infection. Rounded cells following host cell death followed by cell lysis, were observed in cells infected with *R. typhi*^WT^ and *R. typhi*^AZM.^
(TIFF)

S2 Fig Azithromycin resistant strain selection. **Schematic diagram of *R. typhi* strain Wilmington culture using a low concentration of azithromycin (starting at 0.0019 mg/L).** The process was started from the thawing frozen stock of the bacteria and culturing for a week using African green monkey cell line (Vero). Infected cells from the first passage were transferred to fresh cells in a new flask containing 2% RPMI with 0.0019 mg/L of azithromycin and cultured for two generations before increasing the concentration of antibiotic. Starting from an azithromycin concentration of 0.0039 mg/L, the concentration was increased every 4 generations (1 month per concentration). Once the concentration of 0.0625 mg/L was reached the *R. typhi* culture was maintained for 32 generations (~ 8 months). *R. typhi* cultured in media with azithromycin at a concentration of 0.0625 mg/L was kept at -80°C for 1 year. Frozen stock was thawed and inoculated in vero cells. The infected vero cells were pre-grown in media without azithromycin for a week, then sub-cultured to fresh cells maintained in media containing azithromycin concentration of 0.0625 mg/L for 24 generations. The 24^th^ generation of *R. typhi* with azithromycin and the culture without azithromycin, as the control were prepared to determine MIC by plaque assay. The plaque assay was stained with 0.01% of neutral red. The result showed the MIC of azithromycin for *R. typhi*^WT^ was 1 mg/L while MIC for *R. typhi*^AZM^ was > 1 mg/L as shown in Fig 1. Plaques from the assay infected with *R. typhi* with azithromycin at a concentration of 1 mg/L, as well as plaques from the untreated assay, were picked and inoculated into a new culture flask. The *R. typhi*^AZM^ culture was then maintained with azithromycin at a concentration of 0.0625 mg/L for four generations to sustain the strain. The purified plaque of *R. typhi* ^AZM^ was sub-cultured to a new flask of Vero cells and cultured in media containing azithromycin at a concentration of 0.125 mg/L for 8 generations. MIC of azithromycin based on plaque assay for both strains (*R. typhi*^WT^ and *R. typhi*^AZM^) was determined. This schematic was created by the authors.
(TIFF)

S3 Fig Sequence alignment for L4 (*rplD*) and domain V of 23SrRNA. DNA sequence for L4 (a) and Domain V of 23SrRNA (b) from various condition of *R. typhi* culture; *R. typhi*_low passage = *R. typhi* growing for a week after thawing from frozen stock, *R. typhi*_control (*R. typhi*^WT^)= *R. typhi* culture without azithromycin and simultaneously culture with bacteria with azithromycin, *R. typhi*_AZM (*R. typhi*^AZM^) = *R. typhi* culture with low concentration of azithromycin for long period. DNA from the experiment were compared with reference DNA sequence from Kyoto Encyclopedia of Genes and Genomes (KEGG).
PDF)

S4 Fig Protein sequence alignment of L22 protein (*rplV*) from sixteen clinical isolates and nine laboratory strains. L22 amino acid sequence of different strains (TH1527, Wilmington and B9991) from KEGG were also compared.
(PDF)

S5 Fig Sequence alignment for L22 (*rplV).* DNA sequence (a) and amino acids sequence (b) from various conditions of *R. typhi* culture; *R. typhi* low passage = *R. typhi* growing for a week after thawing from frozen stock, *R. typhi* control (*R. typhi*^WT^) = *R. typhi* culture without azithromycin, *R. typhi*^AZM^ = *R. typhi* cultured with low concentration of azithromycin for a long period and *R. typhi*^AZM(-)^ = *R. typhi*^AZM^ cultured in media without azithromycin for 24 generations. These DNA and amino acid sequences were compared with reference DNA sequences from Kyoto Encyclopedia of Genes and Genomes (KEGG).
(PDF)
