# Supplementary material for: A repetitive nucleotide insertion in the rplV gene is associated with in vitro resistance to azithromycin in Rickettsia typhi
Source: PLoS Negl Trop Dis. 2026 Apr 27;20(4):e0014249. doi: 10.1371/journal.pntd.0014249 (PMC13119893; doi:10.1371/journal.pntd.0014249)
Supplement: S1 Table — (DOCX) [file pntd.0014249.s001.docx]

**Supplemental Table**

**Table S1** Macrolide target genes including Domain V of 23SrRNA (DomV), L4 and L22 primers

| **Gene** | **Primers** | **Sequence (5’→ 3’)** | **Product (bp)** |
| --- | --- | --- | --- |
| DomainV of 23SrRNA | Rt-DomV-F | CGATGCTGAAGGAACTAGGC | 1129 |
|  | Rt-DomV-R | GTCTTCCACGGCTCTAATGG |  |
| L4 (*rplD*) | RtyphiL4F2 | TTGATTTGAGTCCCTATAATTCCTTT | 1075 |
|  | RtyphiL4R2 | TTGTGATATTGTGTGAATGTAAAAGC |  |
| L22 (rplV) | RtyphiL22F2 | TTGCGCTTGATTAAACCACTT | 673 |
|  | RtyphiL22R2 | TGTAGGATTTACCTTTTCTGTCCA |  |
