# Supplementary figures and images for: A repetitive nucleotide insertion in the rplV gene is associated with in vitro resistance to azithromycin in Rickettsia typhi
Source: PLoS Negl Trop Dis. 2026 Apr 27;20(4):e0014249. doi: 10.1371/journal.pntd.0014249 (PMC13119893; doi:10.1371/journal.pntd.0014249)

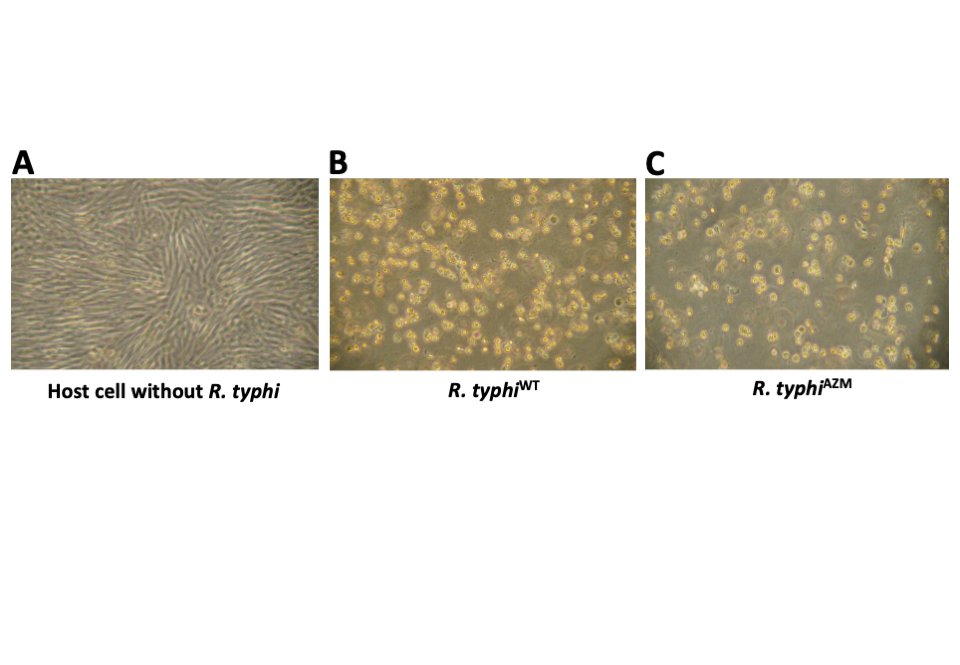

Supplement: S1 Fig — Vero cells were infected with R. typhi strain Wilmington in the culture media without azithromycin (B) and in the low level of azithromycin (C) compared to host cell control (A). CPEs were found both R. typhiWT and R. typhiAZM at Day 7 post infection. Rounded cells following host cell death followed by cell lysis, were observed in cells infected with R. typhiWT and R. typhiAZM. (TIFF) [file pntd.0014249.s002.tiff]

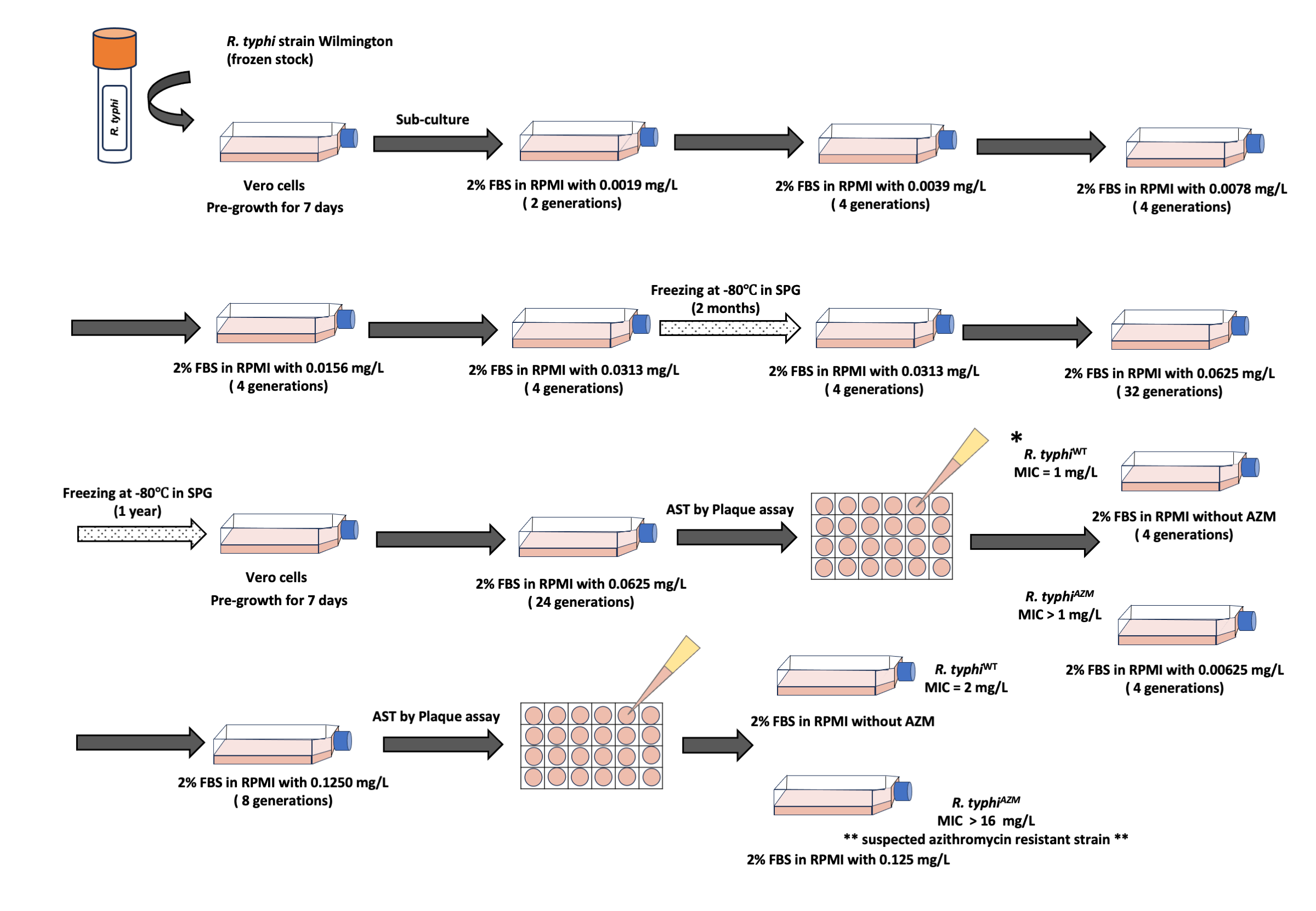

Supplement: S2 Fig — Schematic diagram of R. typhi strain Wilmington culture using a low concentration of azithromycin (starting at 0.0019 mg/L). The process was started from the thawing frozen stock of the bacteria and culturing for a week using African green monkey cell line (Vero). Infected cells from the first passage were transferred to fresh cells in a new flask containing 2% RPMI with 0.0019 mg/L of azithromycin and cultured for two generations before increasing the concentration of antibiotic. Starting from an azithromycin concentration of 0.0039 mg/L, the concentration was increased every 4 generations (1 month per concentration). Once the concentration of 0.0625 mg/L was reached the R. typhi culture was maintained for 32 generations (~ 8 months). R. typhi cultured in media with azithromycin at a concentration of 0.0625 mg/L was kept at -80°C for 1 year. Frozen stock was thawed and inoculated in vero cells. The infected vero cells were pre-grown in media without azithromycin for a week, then sub-cultured to fresh cells maintained in media containing azithromycin concentration of 0.0625 mg/L for 24 generations. The 24th generation of R. typhi with azithromycin and the culture without azithromycin, as the control were prepared to determine MIC by plaque assay. The plaque assay was stained with 0.01% of neutral red. The result showed the MIC of azithromycin for R. typhiWT was 1 mg/L while MIC for R. typhiAZM was > 1 mg/L as shown in Fig 1. Plaques from the assay infected with R. typhi with azithromycin at a concentration of 1 mg/L, as well as plaques from the untreated assay, were picked and inoculated into a new culture flask. The R. typhiAZM culture was then maintained with azithromycin at a concentration of 0.0625 mg/L for four generations to sustain the strain. The purified plaque of R. typhi AZM was sub-cultured to a new flask of Vero cells and cultured in media containing azithromycin at a concentration of 0.125 mg/L for 8 generations. MIC of azithromycin based on plaque ass [file pntd.0014249.s003.tiff]
