# Supplementary material for: A repetitive nucleotide insertion in the rplV gene is associated with in vitro resistance to azithromycin in Rickettsia typhi
Source: PLoS Negl Trop Dis. 2026 Apr 27;20(4):e0014249. doi: 10.1371/journal.pntd.0014249 (PMC13119893; doi:10.1371/journal.pntd.0014249)
Supplement: S3 Fig — DNA sequence for L4 (a) and Domain V of 23SrRNA (b) from various condition of R. typhi culture; R. typhi_low passage = R. typhi growing for a week after thawing from frozen stock, R. typhi_control (R. typhiWT)= R. typhi culture without azithromycin and simultaneously culture with bacteria with azithromycin, R. typhi_AZM (R. typhiAZM) = R. typhi culture with low concentration of azithromycin for long period. DNA from the experiment were compared with reference DNA sequence from Kyoto Encyclopedia of Genes and Genomes (KEGG). PDF) [file pntd.0014249.s004.pdf]

A

```
L4_R.typhi_(RT0650_K02926) ATG AAA ACT AAA ATA TTA AGT CTT GCT AAT GAA GAA GTT GGT GAG ATT ACT TTA AAT AAA GAT ATA TTC GCT GTT GAG  [ 78]
L4_R.typhi_low_passage     ... ... ... ... ... ... ... ... ... ... ... ... ... ... ... ... ... ... ... ... ... ... ... ... ... ...  [ 78]
L4_R._typhi_control        ... ... ... ... ... ... ... ... ... ... ... ... ... ... ... ... ... ... ... ... ... ... ... ... ... ...  [ 78]
L4_R._typhi_AZM            ... ... ... ... ... ... ... ... ... ... ... ... ... ... ... ... ... ... ... ... ... ... ... ... ... ...  [ 78]

L4_R.typhi_(RT0650_K02926) TTT ATC AGA GAT GAT ATA ATA AAG CAG GTT ATC GAT TGG CAG AGA GCT AAA GCA ATG TCC GGT AAC CAT AAA ACT AAA  [156]
L4_R._typhi_low_passage    ... ... ... ... ... ... ... ... ... ... ... ... ... ... ... ... ... ... ... ... ... ... ... ... ... ...  [156]
L4_R._typhi_control        ... ... ... ... ... ... ... ... ... ... ... ... ... ... ... ... ... ... ... ... ... ... ... ... ... ...  [156]
L4_R._typhi_AZM            ... ... ... ... ... ... ... ... ... ... ... ... ... ... ... ... ... ... ... ... ... ... ... ... ... ...  [156]

L4_R.typhi_(RT0650_K02926) ACA GTA TCA GAA GTA TCA GGC ACA ACA AAA AAA CCT TTT AAG CAA AAA GGT ACA GGC AAT GCA CGA CAA GGT TCT CTT  [234]
L4_R._typhi_low_passage    ... ... ... ... ... ... ... ... ... ... ... ... ... ... ... ... ... ... ... ... ... ... ... ... ... ...  [234]
L4_R._typhi_control        ... ... ... ... ... ... ... ... ... ... ... ... ... ... ... ... ... ... ... ... ... ... ... ... ... ...  [234]
L4_R._typhi_AZM            ... ... ... ... ... ... ... ... ... ... ... ... ... ... ... ... ... ... ... ... ... ... ... ... ... ...  [234]

L4_R.typhi_(RT0650_K02926) AGG TCT ATA CAG ATG CGT GGT GGT GGT ATA TCA CAT GGT CCT AAG GTA CGG AGT CAT GCA ATA AAA TTA CCT AAA AAA  [312]
L4_R._typhi_low_passage    ... ... ... ... ... ... ... ... ... ... ... ... ... ... ... ... ... ... ... ... ... ... ... ... ... ...  [312]
L4_R._typhi_control        ... ... ... ... ... ... ... ... ... ... ... ... ... ... ... ... ... ... ... ... ... ... ... ... ... ...  [312]
L4_R._typhi_AZM            ... ... ... ... ... ... ... ... ... ... ... ... ... ... ... ... ... ... ... ... ... ... ... ... ... ...  [312]

L4_R.typhi_(RT0650_K02926) GTA CGA AAA CTT GGT TTA ATT CAT GCT TTA TCT GAG AAA TGT GCT GCA GGA AAA TTA TTA ATA ATA AAT TCT TTA AAG  [390]
L4_R._typhi_low_passage    ... ... ... ... ... ... ... ... ... ... ... ... ... ... ... ... ... ... ... ... ... ... ... ... ... ...  [390]
L4_R._typhi_control        ... ... ... ... ... ... ... ... ... ... ... ... ... ... ... ... ... ... ... ... ... ... ... ... ... ...  [390]
L4_R._typhi_AZM            ... ... ... ... ... ... ... ... ... ... ... ... ... ... ... ... ... ... ... ... ... ... ... ... ... ...  [390]

L4_R.typhi_(RT0650_K02926) TTA GAG AAG CCT AAA ACG TCT GTT CTT GTA AAT TTA TTA AAT AAA TTT CAA GGT CAG AGT TTT TTT ATA ATT GAT GGA  [468]
L4_R._typhi_low_passage    ... ... ... ... ... ... ... ... ... ... ... ... ... ... ... ... ... ... ... ... ... ... ... ... ... ...  [468]
L4_R._typhi_control        ... ... ... ... ... ... ... ... ... ... ... ... ... ... ... ... ... ... ... ... ... ... ... ... ... ...  [468]
L4_R._typhi_AZM            ... ... ... ... ... ... ... ... ... ... ... ... ... ... ... ... ... ... ... ... ... ... ... ... ... ...  [468]

L4_R.typhi_(RT0650_K02926) AAT AAA GTA GAT ACT AAT TTT TCT TTA GCT ACA AAA AAT ATT TAT AAT ACG TTG ATT GTT CCA CAA ATA GGA GCG AAT  [546]
L4_R._typhi_low_passage    ... ... ... ... ... ... ... ... ... ... ... ... ... ... ... ... ... ... ... ... ... ... ... ... ... ...  [546]
L4_R._typhi_control        ... ... ... ... ... ... ... ... ... ... ... ... ... ... ... ... ... ... ... ... ... ... ... ... ... ...  [546]
L4_R._typhi_AZM            ... ... ... ... ... ... ... ... ... ... ... ... ... ... ... ... ... ... ... ... ... ... ... ... ... ...  [546]

L4_R.typhi_(RT0650_K02926) GTT TAT GAT ATC ATA CGA CAT GAG TAT GTA CTC TTA TCA CAA GAA GCT GTG AGC TTT TTA GAA GAG AGG TTA AGA TGA  [624]
L4_R._typhi_low_passage    ... ... ... ... ... ... ... ... ... ... ... ... ... ... ... ... ... ... ... ... ... ... ... ... ... ...  [624]
L4_R._typhi_control        ... ... ... ... ... ... ... ... ... ... ... ... ... ... ... ... ... ... ... ... ... ... ... ... ... ...  [624]
L4_R._typhi_AZM            ... ... ... ... ... ... ... ... ... ... ... ... ... ... ... ... ... ... ... ... ... ... ... ... ... ...  [624]
```

B

```
23SrRNA_R.typhi_(RT0200_K01980) GTA GCG ACT GTT TAT TAA AAA CAC AGG GCT CTG CAA AGT CAA TAG ACG ACG TAT AGG GTC TGA CGC CTG CCC AGT GCT  [ 78]
23SrRNA_R.typhi_low_passage     ... ... ... ... ... ... ... ... ... ... ... ... ... ... ... ... ... ... ... ... ... ... ... ... ... ...  [ 78]
23SrRNA_R.typhi_control         ... ... ... ... ... ... ... ... ... ... ... ... ... ... ... ... ... ... ... ... ... ... ... ... ... ...  [ 78]
23SrRNA_R.typhi_AZM             ... ... ... ... ... ... ... ... ... ... ... ... ... ... ... ... ... ... ... ... ... ... ... ... ... ...  [ 78]

23SrRNA_R.typhi_(RT0200_K01980) GGA AGA TTA AAA GGA GGG GTG CAA GCT CTA AAT TGA AGT CCC AGT GAA CGG CGG CCG TAA CTA TGA CGG TCC TAA GGT  [156]
23SrRNA_R.typhi_low_passage     ... ... ... ... ... ... ... ... ... ... ... ... ... ... ... ... ... ... ... ... ... ... ... ... ... ...  [156]
23SrRNA_R.typhi_control         ... ... ... ... ... ... ... ... ... ... ... ... ... ... ... ... ... ... ... ... ... ... ... ... ... ...  [156]
23SrRNA_R.typhi_AZM             ... ... ... ... ... ... ... ... ... ... ... ... ... ... ... ... ... ... ... ... ... ... ... ... ... ...  [156]

23SrRNA_R.typhi_(RT0200_K01980) AGC GAA ATT CCT TGT CGG GTA AGT TCC GAC CCG CAC GAA TGG CGT AAC GAT TTC CCC ACT GTC TCC AGT ATC GAC TCA  [234]
23SrRNA_R.typhi_low_passage     ... ... ... ... ... ... ... ... ... ... ... ... ... ... ... ... ... ... ... ... ... ... ... ... ... ...  [234]
23SrRNA_R.typhi_control         ... ... ... ... ... ... ... ... ... ... ... ... ... ... ... ... ... ... ... ... ... ... ... ... ... ...  [234]
23SrRNA_R.typhi_AZM             ... ... ... ... ... ... ... ... ... ... ... ... ... ... ... ... ... ... ... ... ... ... ... ... ... ...  [234]

23SrRNA_R.typhi_(RT0200_K01980) GCG AAA TTG AAT TCT CCG TGA AGA TGC GGA GTT CCC GCG GTC AGA CGG AAA GAC CCC GTG AAC CTT TAC TAT AGC TTT  [312]
23SrRNA_R.typhi_low_passage     ... ... ... ... ... ... ... ... ... ... ... ... ... ... ... ... ... ... ... ... ... ... ... ... ... ...  [312]
23SrRNA_R.typhi_control         ... ... ... ... ... ... ... ... ... ... ... ... ... ... ... ... ... ... ... ... ... ... ... ... ... ...  [312]
23SrRNA_R.typhi_AZM             ... ... ... ... ... ... ... ... ... ... ... ... ... ... ... ... ... ... ... ... ... ... ... ... ... ...  [312]

23SrRNA_R.typhi_(RT0200_K01980) GCA CTG GTG TTA GAA GTC AAA TGT GCA GGA TAG GTG GGA GAC TGC GAA GCA GAG GCG TTA GCC TTT GTG GAG TCA CTC  [390]
23SrRNA_R.typhi_low_passage     ... ... ... ... ... ... ... ... ... ... ... ... ... ... ... ... ... ... ... ... ... ... ... ... ... ...  [390]
23SrRNA_R.typhi_control         ... ... ... ... ... ... ... ... ... ... ... ... ... ... ... ... ... ... ... ... ... ... ... ... ... ...  [390]
23SrRNA_R.typhi_AZM             ... ... ... ... ... ... ... ... ... ... ... ... ... ... ... ... ... ... ... ... ... ... ... ... ... ...  [390]

23SrRNA_R.typhi_(RT0200_K01980) TTG AGA TAC CAC CCT TTT GGT TTT TGA TAT CTA ACC GAG ATC CTT GAA TCA GGG TCC GAG ACA ATG CAT GGT GGG TAG  [468]
23SrRNA_R.typhi_low_passage     ... ... ... ... ... ... ... ... ... ... ... ... ... ... ... ... ... ... ... ... ... ... ... ... ... ...  [468]
23SrRNA_R.typhi_control         ... ... ... ... ... ... ... ... ... ... ... ... ... ... ... ... ... ... ... ... ... ... ... ... ... ...  [468]
23SrRNA_R.typhi_AZM             ... ... ... ... ... ... ... ... ... ... ... ... ... ... ... ... ... ... ... ... ... ... ... ... ... ...  [468]

23SrRNA_R.typhi_(RT0200_K01980) TTT GAC TGG GGC GGT CGC CTC CCA AAG AGT AAC GGA GGC GCG CGA TGG TTA GCT CAG GTT GGT CGG AAA TCA ACT TTT  [546]
23SrRNA_R.typhi_low_passage     ... ... ... ... ... ... ... ... ... ... ... ... ... ... ... ... ... ... ... ... ... ... ... ... ... ...  [546]
23SrRNA_R.typhi_control         ... ... ... ... ... ... ... ... ... ... ... ... ... ... ... ... ... ... ... ... ... ... ... ... ... ...  [546]
23SrRNA_R.typhi_AZM             ... ... ... ... ... ... ... ... ... ... ... ... ... ... ... ... ... ... ... ... ... ... ... ... ... ...  [546]

23SrRNA_R.typhi_(RT0200_K01980) AGA GTG CAA TGG CAT AAG CTA GCC TGA CTG CGA GTC TGA CAA GAC GAG CAG AGA CGA AAG TCG GTC ATA GTG ATC CGG  [624]
23SrRNA_R.typhi_low_passage     ... ... ... ... ... ... ... ... ... ... ... ... ... ... ... ... ... ... ... ... ... ... ... ... ... ...  [624]
23SrRNA_R.typhi_control         ... ... ... ... ... ... ... ... ... ... ... ... ... ... ... ... ... ... ... ... ... ... ... ... ... ...  [624]
23SrRNA_R.typhi_AZM             ... ... ... ... ... ... ... ... ... ... ... ... ... ... ... ... ... ... ... ... ... ... ... ... ... ...  [624]

23SrRNA_R.typhi_(RT0200_K01980) TGG TCC CGA GTG GAA GGG CCA TCG CTC AAC GAA TAA AAG GTA CTC CGG GGA TAA CAG GCT GAT GAT TTC CAA GCG TCC  [702]
23SrRNA_R.typhi_low_passage     ... ... ... ... ... ... ... ... ... ... ... ... ... ... ... ... ... ... ... ... ... ... ... ... ... ...  [702]
23SrRNA_R.typhi_control         ... ... ... ... ... ... ... ... ... ... ... ... ... ... ... ... ... ... ... ... ... ... ... ... ... ...  [702]
23SrRNA_R.typhi_AZM             ... ... ... ... ... ... ... ... ... ... ... ... ... ... ... ... ... ... ... ... ... ... ... ... ... ...  [702]

23SrRNA_R.typhi_(RT0200_K01980) ATA GCG ACG AAA TCG TTT GGC ACC TCG ATG TCG GCT CAT CAC ATC CTG GGG CTG GAG AAG GTC CCA AGG GTT CGG CTG  [780]
23SrRNA_R.typhi_low_passage     ... ... ... ... ... ... ... ... ... ... ... ... ... ... ... ... ... ... ... ... ... ... ... ... ... ...  [780]
23SrRNA_R.typhi_control         ... ... ... ... ... ... ... ... ... ... ... ... ... ... ... ... ... ... ... ... ... ... ... ... ... ...  [780]
23SrRNA_R.typhi_AZM             ... ... ... ... ... ... ... ... ... ... ... ... ... ... ... ... ... ... ... ... ... ... ... ... ... ...  [780]

23SrRNA_R.typhi_(RT0200_K01980) TTC GCC GAT TAA AGT GGT ACG TGA GCT GGG TTT AGA ACG TCG TGA GAC AGT TCG GTC CCT ATC TGC CGT GGG TGT AGG  [858]
23SrRNA_R.typhi_low_passage     ... ... ... ... ... ... ... ... ... ... ... ... ... ... ... ... ... ... ... ... ... ... ... ... ... ...  [858]
23SrRNA_R.typhi_control         ... ... ... ... ... ... ... ... ... ... ... ... ... ... ... ... ... ... ... ... ... ... ... ... ... ...  [858]
23SrRNA_R.typhi_AZM             ... ... ... ... ... ... ... ... ... ... ... ... ... ... ... ... ... ... ... ... ... ... ... ... ... ...  [858]

23SrRNA_R.typhi_(RT0200_K01980) AAG TTT GAG AGG ATC TGC CTT TAG TAC GAG AGG ACC GAG GTG GAC GTA CCC CTG GTG GAC CAG TTG TCG TGC CAA CGG  [936]
23SrRNA_R.typhi_low_passage     ... ... ... ... ... ... ... ... ... ... ... ... ... ... ... ... ... ... ... ... ... ... ... ... ... ...  [936]
23SrRNA_R.typhi_control         ... ... ... ... ... ... ... ... ... ... ... ... ... ... ... ... ... ... ... ... ... ... ... ... ... ...  [936]
23SrRNA_R.typhi_AZM             ... ... ... ... ... ... ... ... ... ... ... ... ... ... ... ... ... ... ... ... ... ... ... ... ... ...  [936]

23SrRNA_R._typhi_(RT0200_K01980) CAC AGC TGG GTA GCT AAG  [954]
23SrRNA_R._typhi_low_passage     ... ... ... ... ... ...  [954]
23SrRNA_R._typhi_control         ... ... ... ... ... ...  [954]
23SrRNA_R.typhi_AZM              ... ... ... ... ... ...  [954]
```
