# Supplementary material for: A repetitive nucleotide insertion in the rplV gene is associated with in vitro resistance to azithromycin in Rickettsia typhi
Source: PLoS Negl Trop Dis. 2026 Apr 27;20(4):e0014249. doi: 10.1371/journal.pntd.0014249 (PMC13119893; doi:10.1371/journal.pntd.0014249)
Supplement: S4 Fig — L22 amino acid sequence of different strains (TH1527, Wilmington and B9991) from KEGG were also compared. (PDF) [file pntd.0014249.s005.pdf]

```
L22_R._typhi_Wilmington .......... .......... .......... .......... .......... ..........  [ 60]
L22_R._typhi_B9991      .......... .......... .......... .......... .......... ..........  [ 60]
L22-AZ306               .......... .......... .......... .......... .......... ..........  [ 60]
L22-AZ331               .......... .......... .......... .......... .......... ..........  [ 60]
L22-FLA6950             .......... .......... .......... .......... .......... ..........  [ 60]
L22-GEAR                .......... .......... .......... .......... .......... ..........  [ 60]
L22-GER                 .......... .......... .......... .......... .......... ..........  [ 60]
L22-Musseibov           .......... .......... .......... .......... .......... ..........  [ 60]
L22-NA16PP              .......... .......... .......... .......... .......... ..........  [ 60]
L22-TA837               .......... .......... .......... .......... .......... ..........  [ 60]
L22-Wilmington          .......... .......... .......... .......... .......... ..........  [ 60]
L22-TM1041              .......... .......... .......... .......... .......... ..........  [ 60]
L22-TM1377              .......... .......... .......... .......... .......... ..........  [ 60]
L22-TM2418              .......... .......... .......... .......... .......... ..........  [ 60]
L22-TM2504              .......... .......... .......... .......... .......... ..........  [ 60]
L22-TM2522              .......... .......... .......... .......... .......... ..........  [ 60]
L22-TM2529              .......... .......... .......... .......... .......... ..........  [ 60]
L22-TM2540              .......... .......... .......... .......... .......... ..........  [ 60]
L22-TM2557              .......... .......... .......... .......... .......... ..........  [ 60]
L22-TM3627              .......... .......... .......... .......... .......... ..........  [ 60]
L22-TM3905              .......... .......... .......... .......... .......... ..........  [ 60]
L22-TM4034              .......... .......... .......... .......... .......... ..........  [ 60]
L22-TM4105              .......... .......... .......... .......... .......... ..........  [ 60]
L22-TM4234              .......... .......... .......... .......... .......... ..........  [ 60]
L22-TM4237              .......... .......... .......... .......... .......... ..........  [ 60]
L22-TM8956              .......... .......... .......... .......... .......... ..........  [ 60]
L22-TM10184             .......... .......... .......... .......... .......... ..........  [ 60]

L22_R._typhi_TH1527     VANAENNLGL DIDRLIITKA TVGKSVVMKR IMPRAKGRAT RINKFFSNLD ITVTEKEDN*  [120]
L22_R._typhi_Wilmington .......... .......... .......... .......... .......... ..........  [120]
L22_R._typhi_B9991      .......... .......... .......... .......... .......... ..........  [120]
L22-AZ306               .......... .......... .......... .......... .......... ..........  [120]
L22-AZ331               .......... .......... .......... .......... .......... ..........  [120]
L22-FLA6950             .......... .......... .......... .......... .......... ..........  [120]
L22-GEAR                .......... .......... .......... .......... .......... ..........  [120]
L22-GER                 .......... .......... .......... .......... .......... ..........  [120]
L22-Musseibov           .......... .......... .......... .......... .......... ..........  [120]
L22-NA16PP              .......... .......... .......... .......... .......... ..........  [120]
L22-TA837               .......... .......... .......... .......... .......... ..........  [120]
L22-Wilmington          .......... .......... .......... .......... .......... ..........  [120]
L22-TM1041              .......... .......... .......... .......... .......... ..........  [120]
L22-TM1377              .......... .......... .......... .......... .......... ..........  [120]
L22-TM2418              .......... .......... .......... .......... .......... ..........  [120]
L22-TM2504              .......... .......... .......... .......... .......... ..........  [120]
L22-TM2522              .......... .......... .......... .......... .......... ..........  [120]
L22-TM2529              .......... .......... .......... .......... .......... ..........  [120]
L22-TM2540              .......... .......... .......... .......... .......... ..........  [120]
L22-TM2557              .......... .......... .......... .......... .......... ..........  [120]
L22-TM3627              .......... .......... .......... .......... .......... ..........  [120]
L22-TM3905              .......... .......... .......... .......... .......... ..........  [120]
L22-TM4034              .......... .......... .......... .......... .......... ..........  [120]
L22-TM4105              .......... .......... .......... .......... .......... ..........  [120]
L22-TM4234              .......... .......... .......... .......... .......... ..........  [120]
L22-TM4237              .......... .......... .......... .......... .......... ..........  [120]
L22-TM8956              .......... .......... .......... .......... .......... ..........  [120]
L22-TM10184             .......... .......... .......... .......... .......... ..........  [120]
```
