# Supplementary material for: A repetitive nucleotide insertion in the rplV gene is associated with in vitro resistance to azithromycin in Rickettsia typhi
Source: PLoS Negl Trop Dis. 2026 Apr 27;20(4):e0014249. doi: 10.1371/journal.pntd.0014249 (PMC13119893; doi:10.1371/journal.pntd.0014249)
Supplement: S5 Fig — DNA sequence (a) and amino acids sequence (b) from various conditions of R. typhi culture; R. typhi low passage = R. typhi growing for a week after thawing from frozen stock, R. typhi control (R. typhiWT) = R. typhi culture without azithromycin, R. typhiAZM = R. typhi cultured with low concentration of azithromycin for a long period and R. typhiAZM(-) = R. typhiAZM cultured in media without azithromycin for 24 generations. These DNA and amino acid sequences were compared with reference DNA sequences from Kyoto Encyclopedia of Genes and Genomes (KEGG). (PDF) [file pntd.0014249.s006.pdf]

A

```
R._typhi_(RT0646_K02890) ATG ATA CAG GAA AAT AAA AAT TTT GCT ACA GCA AAA GCT AAG TCT ATT AGA GTA AGT CCA AGG AAG CTT  [ 69]
R._typhi_low_passage     ... ... ... ... ... ... ... ... ... ... ... ... ... ... ... ... ... ... ... ... ... ... ...  [ 69]
R._typhi_control         ... ... ... ... ... ... ... ... ... ... ... ... ... ... ... ... ... ... ... ... ... ... ...  [ 69]
R._typhi_AZM             ... ... ... ... ... ... ... ... ... ... ... ... ... ... ... ... ... ... ... ... ... ... ...  [ 69]
R._typhi_AZM(-)          ... ... ... ... ... ... ... ... ... ... ... ... ... ... ... ... ... ... ... ... ... ... ...  [ 69]

R._typhi_(RT0646_K02890) AAT TTA GTT GCC TCC TTT ATT AGA AAT ATG AAA GTA TCT GAA GCA TTA ATA CAA TTA ACT TTT TCT CCT  [138]
R._typhi_low_passage     ... ... ... ... ... ... ... ... ... ... ... ... ... ... ... ... ... ... ... ... ... ... ...  [138]
R._typhi_control         ... ... ... ... ... ... ... ... ... ... ... ... ... ... ... ... ... ... ... ... ... ... ...  [138]
R._typhi_AZM             ... ... ... ... ... ... ... ... ... ... ... ... ... ... ... ... ... ... ... ... ... ... ...  [138]
R._typhi_AZM(-)          ... ... ... ... ... ... ... ... ... ... ... ... ... ... ... ... ... ... ... ... ... ... ...  [138]

R._typhi_(RT0646_K02890) AAA AGA ATT GCA AAA ATT GTA AAA GAT TGT TTA CGA TCT GCT GTT GCA AAT GCT GAA AAT AAT TTA GGT  [207]
R._typhi_low_passage     ... ... ... ... ... ... ... ... ... ... ... ... ... ... ... ... ... ... ... ... ... ... ...  [207]
R._typhi_control         ... ... ... ... ... ... ... ... ... ... ... ... ... ... ... ... ... ... ... ... ... ... ...  [207]
R._typhi_AZM             ... ... ... ... ... ... ... ... ... ... ... ... ... ... ... ... ... ... ... ... ... ... ...  [207]
R._typhi_AZM(-)          ... ... ... ... ... ... ... ... ... ... ... ... ... ... ... ... ... ... ... ... ... ... ...  [207]

R._typhi_(RT0646_K02890) TTA GAC ATA GAT AGG TTA ATT ATT ACT AAA GCT ACT GTA GGT AAG TCG GTC GTA ATG AAA AGG ATT ATG  [276]
R._typhi_low_passage     ... ... ... ... ... ... ... ... ... ... ... ... ... ... ... ... ... ... ... ... ... ... ...  [276]
R._typhi_control         ... ... ... ... ... ... ... ... ... ... ... ... ... ... ... ... ... ... ... ... ... ... ...  [276]
R._typhi_AZM             ... ... ... ... ... ... ... ... ... ... ... ... ... ... ... ... ... ... ... ... ... ... ...  [276]
R._typhi_AZM(-)          ... ... ... ... ... ... ... ... ... ... ... ... ... ... ... ... ... ... ... ... ... ... ...  [276]

R._typhi_(RT0646_K02890) CCG AGA GCA --- --- --- --- --- AAA GGA AGA GCA ACT AGA ATA AAT AAG TTT TTT AGT AAT CTT GAT  [345]
R._typhi_low_passage     ... ... ... --- --- --- --- --- ... ... ... ... ... ... ... ... ... ... ... ... ... ... ...  [345]
R._typhi_control         ... ... ... --- --- --- --- --- ... ... ... ... ... ... ... ... ... ... ... ... ... ... ...  [345]
R._typhi_AZM             ... ... ... AAA GGA AGA GCA ACT ... ... ... ... ... ... ... ... ... ... ... ... ... ... ...  [345]
R._typhi_AZM(-)          ... ... ... --- --- --- --- --- ... ... ... ... ... ... ... ... ... ... ... ... ... ... ...  [345]

R._typhi_(RT0646_K02890) ATA ACT GTT ACA GAA AAA GAG GAT AAT TAA  [375]
R._typhi_low_passage     ... ... ... ... ... ... ... ... ... ...  [375]
R._typhi_control         ... ... ... ... ... ... ... ... ... ...  [375]
R._typhi_AZM             ... ... ... ... ... ... ... ... ... ...  [375]
R._typhi_AZM(-)          ... ... ... ... ... ... ... ... ... ...  [375]
```

B

```
R._typhi_(RT0646_K02890) MIQENKNFAT AKAKSIRVSP RKLNLVASFI RNMKVSEALI QLTFSPKRIA KIVKDCLRSA VANAENNLGL  [ 70]
R._typhi_low_passage     .......... .......... .......... .......... .......... .......... ..........  [ 70]
R._typhi_control         .......... .......... .......... .......... .......... .......... ..........  [ 70]
R._typhi_AZM             .......... .......... .......... .......... .......... .......... ..........  [ 70]
R._typhi_AZM(-)          .......... .......... .......... .......... .......... .......... ..........  [ 70]

R._typhi_(RT0646_K02890) DIDRLIITKA TVGKSVVMKR IMPRA----- KGRATRINKF FSNLDITVTE KEDN* [125]
R._typhi_low_passage     .......... .......... .....----- .......... .......... ..... [125]
R._typhi_control         .......... .......... .....----- .......... .......... ..... [125]
R._typhi_AZM             .......... .......... .....KGRAT .......... .......... ..... [125]
R._typhi_AZM(-)          .......... .......... .....----- .......... .......... ..... [125]
```
